# Validation List no. 226: valid publication of new names and new combinations effectively published outside the IJSEM

**DOI:** 10.1099/ijsem.0.006910

**Published:** 2025-12-12

**Authors:** Aharon Oren, Markus Göker

**Affiliations:** 1 The Institute of Life Sciences, The Hebrew University of Jerusalem, The Edmond J. Safra Campus, 9190401 Jerusalem, Israel; 2 Leibniz Institute DSMZ – German Collection of Microorganisms and Cell Cultures, Inhoffenstrasse 7B, 38124 Braunschweig, Germany

The purpose of this list is to publish in the International Journal of Systematic and Evolutionary Microbiology (IJSEM) those new names and new combinations that were effectively published outside the IJSEM as set forth in Rule 27 of the International Code of Nomenclature of Prokaryotes (ICNP; 2022 Revision) [1]. Authors and other individuals wishing to have new names and/or combinations included in future lists should send an electronic copy of the published paper to the IJSEM Editorial Office for confirmation that all of the other requirements for valid publication have been met. It is also a requirement of IJSEM and the ICNP that authors of new species, new subspecies and new combinations provide evidence that types are deposited in two publicly accessible culture collections in two different countries. It should be noted that the date of valid publication of these new names and combinations is the date of publication of this list, not the date of the original publication of the names and combinations. The authors of the new names and combinations are as given below.

Inclusion of a name on these lists validates the publication of the name and thereby makes it available in the nomenclature of prokaryotes. Inclusion of a name on these lists is part of the requirements of valid publication of an effectively published name. Indeed, some of these names may, in time, be considered later synonyms, or it may be proposed to transfer the organisms to another genus, thus necessitating the creation of a new combination. Conversely, it may be proposed to transfer an organism back to an earlier genus and to regard the basonym or an earlier new combination as the correct name instead of a more recent new combination. The ICNP does not decide on such taxonomic opinions.

**Table:** 

Name/authors	Proposed as	Nomenclatural type^1^	Priority^2^	Reference
*Absicoccus intestinalis* Hitch *et al*. 2024, 6^3,4^	sp. nov.	CLA-KB-P134 (=DSM 114836=JCM 37183)	90	[2]
*Acinetobacter oryzae* Chaudhary *et al*. 2012, 820	sp. nov.	B23 (=CGMCC 1.10689=LMG 25575)	10	[3]
*Acinetobacter plantarum* Du *et al*. 2016, 396	sp. nov.	THG-SQM11 (=CCTCC AB 2015123=KCTC 42611)	71	[4]
*Aliihoeflea* Roh *et al*. 2008, 596	gen. nov.	*Aliihoeflea aestuarii*	11	[5]
*Aliihoeflea aestuarii* Roh *et al*. 2008, 596	sp. nov.	N8 (=DSM 19536=JCM 15118=KCTC 22052)	11	[5]
*Aliiphycobium* Kim *et al*. 2025, 9^3^	gen. nov.	*Aliiphycobium algicola*	40	[6]
*Aliiphycobium algicola* Kim *et al*. 2025, 9^3^	sp. nov.	G2-2 (=JCM 35752=KACC 22602)	40	[6]
*Aliisedimentitalea* Kim *et al*. 2015, 500	gen. nov.	*Aliisedimentitalea scapharcae*	141	[7]
*Aliisedimentitalea scapharcae* Kim *et al*. 2015, 501	sp. nov.	MA2-16 (=CECT 8598=KCTC 42119)	141	[7]
*Alistipes montrealensis* Routy *et al*. 2022, 149^5^	sp. nov.	kh20 (=CECT 30384=CSUR Q6005)	46	[8]
*Alkalihalobacillus phoenicis* Schultz *et al*. 2025, 8^3^	sp. nov.	1P02AB (=DSM 118404=MTCC 13926)	4	[9]
*Ancylomarina euxina* corrig. Yadav *et al*. 2020, 8^3,6^	sp. nov.	M1P (=JCM 32614=KCTC 15718)	35	[10]
*Anoxybacillus kamchatkensis* subsp. *asaccharedens* Gul-Guven *et al*. 2008, 333	subsp. nov.	KG8 (=CIP 109280=DSM 18475)	103	[11]
*Anoxybacillus kamchatkensis* subsp. *kamchatkensis* (Kevbrin *et al*. 2006) Gul-Guven *et al*. 2008, 333^7^	comb. nov. [basonym: *Anoxybacillus kamchatkensis* Kevbrin *et al*. 2006]	JW-VK-KG4 (=ATCC BAA-549=DSM 14988)	103	[11]
*Arthrobacter phoenicis* Schultz *et al*. 2025, 9^3^	sp. nov.	1P04PC (=DSM 117984=MTCC 13669)	4	[9]
*Aureimonas fodinaquatilis* Baek *et al*. 2020, 2658	sp. nov.	CAU 1482 (=KCTC 62995=NBRC 113692)	129	[12]
*Bacillus hunanensis* Chen *et al*. 2011, 486^8^	sp. nov.	JSM 081003 (=DSM 23008=KCTC 13711)	28	[13]
*Bacillus jepli* Schultz *et al*. 2025, 9^3^	sp. nov.	1P02SD (=DSM 117993=MTCC 13928)	4	[9]
*Bacillus kalamii* Schultz *et al*. 2025, 10^3^	sp. nov.	1P06AnD (=DSM 117991=MTCC 13638)	4	[9]
*Bacillus multifaciens* Luo *et al*. 2025, 14^3^	sp. nov.	WLY-B-L8 (=CICC 25210=JCM 36284)	21	[14]
*Bacteroides celer* Yang *et al*. 2025, 7^3^	sp. nov.	KFT8 (=JCM 36011=KCTC 15614)^9^	34	[15]
*Bacteroides mucinivorans* Yang *et al*. 2025, 9^3^	sp. nov.	CG01 (=JCM 36010=KCTC 15613)	34	[15]
*Binariimonas* Zhao *et al*. 2016, 280^10^	gen. nov.	*Binariimonas pacifica*	49	[16]
*Binariimonas pacifica* Zhao *et al*. 2016, 280^11^	sp. nov.	JLT 2480 (=CGMCC 1.12850=DSM 28646)	49	[16]
*Bisbaumannia salifodinae* (Wang *et al*. 2008) León-Lemus *et al*. 2025, 15^3^	comb. nov. [basonym: *Halomonas salifodinae* Wang *et al*. 2008]	BC7 (=CGMCC 1.6774=JCM 14803)	127	[17]
*Brevundimonas phoenicis* Schultz *et al*. 2025, 13^3^	sp. nov.	2P2-tot-C (=DSM 118058=MTCC 13894)	4	[9]
*Caldimonas hydrothermalis* corrig. Bouraoui *et al*. 2010, 490^12^	sp. nov.	HAN-85 (=DSM 18497=LMG 23755)	42	[18]
*Caldimonas mangrovi* Yang *et al*. 2023, 505	sp. nov.	S2-27 (=CGMCC 4.7715=JCM 34532)	47	[19]
*Capnocytophaga endodontalis* Jo *et al*. 2018, 423	sp. nov.	ChDC OS43 (=JCM 32133=KCTC 5562)^13^	114	[20]
*Caproiciproducens faecalis* Choi *et al*. 2023, 5^3^	sp. nov.	AGMB10547 (=GDMCC 1.2575=KCTC 25200)^14^	88	[21]
*Catellatospora aurea* Liu *et al*. 2014, 1189	sp. nov.	NEAU-SH16 (=CGMCC 4.7147=DSM 46719)	6	[22]
*Cellulosispirillum* Sorokin *et al*. 2025, 7^3^	gen. nov.	*Cellulosispirillum alkaliphilum*	41	[23]
*Cellulosispirillum alkaliphilum* Sorokin *et al*. 2025, 7^3^	sp. nov.	ANBcel5 (=DSM 113825=UQM 41584)^15^	41	[23]
*Chelativorans alearensis* corrig. Meng *et al*. 2021, 1660^16^	sp. nov.	UJN715 (=CCTCC AB 2019378=KCTC 72856)	77	[24]
*Chitinibacter suncheonensis* Kim *et al*. 2012, 1061^17^	sp. nov.	SK16 (=DSM 25421=KCTC 23839)	14	[25]
*Chryseobacterium zhengzhouense* Wang *et al*. 2016, 1304	sp. nov.	M05W1-39A1 (=CGMCC 1.15067=JCM 30863)^18^	86	[26]
*Cohnella abietis* Jiang *et al*. 2019, 957	sp. nov.	HS21 (=CCTCC AB 2019010=KCTC 43028)	18	[27]
*Cohnella candida* corrig. Maeng *et al*. 2019, 1035^19^	sp. nov.	18JY8-7 (=JCM 33199=KCTC 33969)	115	[28]
*Congregicoccus* Islam *et al*. 2025, 11^3^	gen. nov.	*Congregicoccus parvus*	33	[29]
*Congregicoccus parvus* Islam *et al*. 2025, 11^3^	sp. nov.	ASA1 (=JCM 36569=NCIMB 15508)	33	[29]
*Corallincola luteus* Ren *et al*. 2019, 1696	sp. nov.	DASS28 (=KCTC 52376=MCCC 1K03208)	101	[30]
*Cupriavidus consociatus* Tapia-García *et al*. 2025, 12^3^	sp. nov.	LEh25 (=ATCC TSD-314=CDBB B-2085)^20^	23	[31]
*Curtobacterium phoenicis* Schultz *et al*. 2025, 12^3^	sp. nov.	1P10AnD (=DSM 117985=MTCC 13639)	4	[9]
*Deinococcus saharensis* corrig. Bouraoui *et al*. 2012, 320^21^	sp. nov.	HAN23 (=DSM 18496=LMG 23756)^22^	27	[32]
*Desertivibrio* Li *et al*. 2025, 12^3^	gen. nov.	*Desertivibrio insolitus*	59	[33]
*Desertivibrio insolitus* Li *et al*. 2025, 12^3^	sp. nov.	SYSU D00978 (=CGMCC 1.18633=KCTC 49492=MCCC 1K04997)	59	[33]
*Desulfococcus niacini* Imhoff-Stuckle and Pfennig 1983, 197^23^	sp. nov.	NAV-1 (=DSM 2650=JCM 12294)	142	[34]
*Elusimicrobium posterum* Mies *et al*. 2025, 12^3^	sp. nov.	Dip510 (=DSM 104965=JCM 31828)	19	[35]
*Elusimicrobium simillimum* Mies *et al*. 2025, 12^3^	sp. nov.	Sma112 (=DSM 104966=JCM 31830)	19	[35]
*Ewingella docleensis* Prieto *et al*. 2025, 9^3^	sp. nov.	CoE-038–23 (=DSM 117356=NCIMB 15527)	126	[36]
*Fibrisoma montanum* Huang *et al*. 2020, 272	sp. nov.	HYT19 (=CCTCC AB 2018342=JCM 33105)	80	[37]
*Flavitalea flava* Liu *et al*. 2019, 280	sp. nov.	AN120636 (=CCTCC AB 2017174=KCTC 52346)	69	[38]
*Flavobacterium okayamense* Kitahara *et al*. 2023, 5^3^	sp. nov.	KK2020170 (=ATCC TSD-280=NBRC 115344)	110	[39]
*Flavobacterium shanxiense* Yang *et al*. 2015, 837	sp. nov.	YF-2 (=CCTCC AB 2014079=JCM 30153)	75	[40]
*Fluviispira vulneris* Tang *et al*. 2023, 1314	sp. nov.	GX5518 (=CGMCC 1.18685=KCTC 82149)	55	[41]
*Geobacillus caldiproteolyticus* corrig. Chen *et al*. 2004, 496^24^	sp. nov.	SF03 (=ATCC BAA-818=DSM 15730=LMG 26209)	24	[42]
*Geobacillus thermoleovorans* subsp. *stromboliensis* Romano *et al*. 2005, 188^25^	subsp. nov.	Pizzo (=ATCC BAA-979=DSM 15392)	140	[43]
*Geobacillus thermoleovorans* subsp. *thermoleovorans* (Nazina *et al*. 2001) Romano *et al*. 2005, 188^26^	comb. nov. [basonym: *Geobacillus thermoleovorans* Nazina *et al*. 2001]	LEH-1 (=ATCC 43513=BGSG 96A1=DSM 5366=LMG 9823)	140	[43]
*Georgenia faecalis* Wang *et al*. 2020, 739	sp. nov.	ZLJ0423 (=CGMCC 1.13681=JCM 33470)	119	[44]
*Georgenia phoenicis* Schultz *et al*. 2025, 8^3^	sp. nov.	1P01AC (=DSM 117974=MTCC 13825)	4	[9]
*Glycomyces xinjiangensis* Guan *et al*. 2017, 1234^27^	sp. nov.	XHU 5301 (=CCTCC AA 2016043=KCTC 39689)	139	[45]
*Grylomicrobium* Hitch *et al*. 2024, 4^3,28^	gen. nov.	*Grylomicrobium aquisgranense* corrig.	90	[2]
*Grylomicrobium aquisgranense* corrig. Hitch *et al*. 2024, 4^3,29^	sp. nov.	CLA-KB-P133 (=DSM 114835=JCM 37182)	90	[2]
*Halobacterium yunchengense* Cui *et al*. 2024, 6^3^	sp. nov.	YCN1 (=CGMCC 1.62711=KCTC 4334=MCCC 4K00190)	145	[46]
*Halobellus salinisoli* Cui *et al*. 2024, 8^3^	sp. nov.	LT62 (=CGMCC 1.62741=KCTC 4331=MCCC 4K00183)	145	[46]
*Halocynthiibacter halioticoli* Zhang *et al*. 2023, 7^3^	sp. nov.	Z654 (=KCTC 92003=MCCC 1H00503)	134	[47]
*Halorientalis halophila* Cui *et al*. 2024, 7^3^	sp. nov.	LT38 (=CGMCC 1.62710=KCTC 4333=MCCC 4K00199)	145	[46]
*Halosimplex amylolyticum* Mao *et al*. 2025, 8^3^	sp. nov.	DYHT-AS-1 (=CGMCC 1.62752=JCM 37371)	2	[48]
*Halosimplex halobium* Mao *et al*. 2025, 8^3^	sp. nov.	GDY60 (=CGMCC 1.18546=JCM 34482)	2	[48]
*Halosimplex marinum* Mao *et al*. 2025, 9^3^	sp. nov.	XH63 (=CGMCC 1.17474=JCM 34315)	2	[48]
*Halosimplex rarum* Mao *et al*. 2025, 9^3^	sp. nov.	TS25 (=KCTC 4309=MCCC 4K00131)	2	[48]
*Haloterrigena gelatinilytica* Liu *et al*. 2022, 6^3^	sp. nov.	SYSU A558-1 (=CGMCC 1.15953=KCTC 4259)	50	[49]
*Halovenus amylolytica* Mao *et al*. 2025, 10^3^	sp. nov.	SHR40 (=CGMCC 1.14961=JCM 30833)	2	[48]
*Halovenus halobia* Mao *et al*. 2025, 10^3^	sp. nov.	ZY30 (=CGMCC 1.17179=JCM 34159)	2	[48]
*Halovenus marina* Mao *et al*. 2025, 10^3^	sp. nov.	SYNS179 (=CGMCC 1.62726=JCM 37151)	2	[48]
*Hanstruepera flava* Ding *et al*. 2022, 10^3^	sp. nov.	NBU2984 (=KCTC 92511=MCCC 1K07472)	112	[50]
*Hymenobacter guriensis* Damdintogtokh *et al*. 2021, 3339	sp. nov.	BT594 (=KCTC 21863=NBRC 114853)	36	[51]
*Hymenobacter properus* Bang *et al*. 2021, 1137	sp. nov.	BT439 (=KCTC 72900=NBRC 114849)	132	[52]
*Hymenobacter translucens* Bang *et al*. 2022, 5^3^	sp. nov.	BT175 (=KCTC 72330=NBRC 115441)	113	[53]
*Hymenobacter puniceus* Park *et al*. 2021, 1651	sp. nov.	BT190 (=KCTC 72342=NBRC 114860)	117	[54]
*Intestinicryptomonadaceae* corrig. Hitch *et al*. 2024, 3^3,30^	fam. nov.	*Intestinicryptomonas*	90	[2]
*Intestinicryptomonas* Hitch *et al*. 2024, 3^3^	gen. nov.	*Intestinicryptomonas porci*	90	[2]
*Intestinicryptomonas porci* Hitch *et al*. 2024, 3^3^	sp. nov.	CLA-KB-P66 (=DSM 114844=JCM 37181)	90	[2]
*Isoptericola salitolerans* Guan *et al*. 2013, 473	sp. nov.	TRM F109 (=JCM 15901=KCTC 19617)	12	[55]
*Jannaschia halovivens* Yoon and Oh 2025, 7^3^	sp. nov.	KMU-145 (=KCCM 90597=NBRC 117092)	87	[56]
*Jannaschia ovalis* Kwon *et al*. 2025, 10^3^	sp. nov.	GRR-S6-38 (=JCM 36187=KACC 22240=KCTC 82518)	111	[57]
*Jejuibacter* corrig. Jiang *et al*. 2020, 364^31^	gen. nov.	*Jejuibacter calystegiae* corrig.	76	[58]
*Jejuibacter calystegiae* corrig. Jiang *et al*. 2020, 364	sp. nov.	KSNA2 (=CCTCC AB 2019098=KCTC 72234)	76	[58]
*Kangiella chungangensis* Kim *et al*. 2015, 1296	sp. nov.	CAU 1040 (=KCTC 42299=NBRC 110728)	99	[59]
*Kiloniella litopenaei* Wang *et al*. 2015, 1298	sp. nov.	P1-1 (=LMG 27755=MCCC 1A09869)	124	[60]
*Kitasatospora grisea* Nakamura *et al*. 1989, 26^32^	sp. nov.	AA-107 (=ATCC 49232=JCM 7249=NBRC 14835)^33^	131	[61]
*Kitasatospora papulosa* Nakamura *et al*. 1989, 26^32^	sp. nov.	AB-110 (=ATCC 49231=DSM 41643=JCM 7250=NBRC 14834)^34^	131	[61]
*Kocuria kalidii* Xu *et al*. 2025, 6^3^	sp. nov.	M4R5S9 (=CGMCC 1.64776=JCM 37381)	108	[62]
*Kocuria rhizosphaerae* Xu *et al*. 2025, 6^3^	sp. nov.	M1R5S2 (=CGMCC 1.64778=JCM 37379)	108	[62]
*Kocuria rhizosphaericola* Xu *et al*. 2025, 6^3^	sp. nov.	M4R2S49 (=CGMCC 1.64777=JCM 37380)	108	[62]
*Kolteria* Kallscheuer *et al*. 2025, 16^3^	gen. nov.	*Kolteria novifilia*	92	[63]
*Kolteria novifilia* Kallscheuer *et al*. 2025, 16^3^	sp. nov.	Pan216 (=CECT 9536=DSM 100414)	92	[63]
*Kolteriaceae* Kallscheuer *et al*. 2025, 16^3^	fam. nov.	*Kolteria*	92	[63]
*Kolteriales* Kallscheuer *et al*. 2025, 16^3^	ord. nov.	*Kolteria*	92	[63]
*Lacticaseibacillus suihuaensis* Zhu *et al*. 2025, 7^3^	sp. nov.	N149-9 (=CCTCC AB 2024126=JCM 36998=LMG 33660)	61	[64]
*Liberiplasma* Khomyakova *et al*. 2025, 10^3^	gen. nov.	*Liberiplasma polymorphum*	26	[65]
*Liberiplasma polymorphum* Khomyakova *et al*. 2025, 10^3^	sp. nov.	C05PYG (=BIM B-2060=VKM B-3767)^35^	26	[65]
*Liberiplasmataceae* Khomyakova *et al*. 2025, 10^3^	fam. nov.	*Liberiplasma*	26	[65]
*Liberiplasmatales* Khomyakova *et al*. 2025, 10^3^	ord. nov.	*Liberiplasma*	26	[65]
*Luteibacter pinisoli* Akter and Huq 2018, 1021	sp. nov.	MAH-14 (=CGMCC 1.16227=KACC 19298)	89	[66]
*Luteimonas viscosa* Chen *et al*. 2022, 758	sp. nov.	XBU10 (=CGMCC 1.12158=KCTC 23878)	118	[67]
*Lysinibacillus canaveralius* Schultz *et al*. 2025, 13^3^	sp. nov.	3P01SB (=DSM 118032=MTCC 13643)	4	[9]
*Lysinibacillus phoenicis* Schultz *et al*. 2025, 8^3^	sp. nov.	1P01SD (=DSM 117981=MTCC 13636)	4	[9]
*Lysobacter cookii* (Christensen 1978) Kawamura *et al*. 2009, 122	comb. nov. [basonym: *Lysobacter enzymogenes* subsp. *cookii* Christensen 1978]	PAGU 1119 (= ATCC 29488=JCM 16562)	32	[68]
*Lysobacter gilvus* Xu *et al*. 2021, 10	sp. nov.	HX-5–24 (=CCTCC AB 2019228=KCTC 72470)	78	[69]
*Lysobacter lactosilyticus* Wang *et al*. 2023, 5^3^	sp. nov.	A6 (=CGMCC 1.18582=KCTC 82184)	100	[70]
*Marinobacter piscis* corrig. Hedi *et al*. 2015, 548^36^	sp. nov.	Abdou2 (=CCUG 63769=DSM 26804)^37^	107	[71]
*Marinobacter squalenivorans* Rontani *et al*. 2003, 4173^38^	sp. nov.	2A sq64 (=ATCC BAA-792=DSM 15125)^39^	9	[72]
*Maritimibacter alexandrii* Wang *et al*. 2021, 4002	sp. nov.	LZ-17 (=CCTCC 2019005=KCTC 72193)	84	[73]
*Maritimibacter harenae* Khan *et al*. 2021, 808	sp. nov.	DP07 (=JCM 33811=KACC 21429)	30	[74]
*Massilia humi* Du and Yin 2016, 366	sp. nov.	THG-S6.8 (=CCTCC AB 2015296=KCTC 42737)	74	[75]
*Mesoplasma whartonense* Green *et al*. 2025, 21^3,40^	sp. nov.	JKS002660 (=DSM 115716=NCTC 14863)^39^	45	[76]
*Mesorhizobium denitrificans* Siddiqi *et al*. 2019, 241	sp. nov.	LA-28 (=KACC 19675=LMG 30806)	122	[77]
*Microbacterium canaveralium* Schultz *et al*. 2025, 11^3^	sp. nov.	1P10AE (=DSM 117987=MTCC 13670)	4	[9]
*Microbacterium mcarthurae* Hill *et al*. 2025, 5^3^	sp. nov.	1F8SW-P5 (=DSM 115934=NRRL B-65667)	93	[78]
*Microbacterium pratiae* Schultz *et al*. 2025, 13^3^	sp. nov.	2P06AB (=DSM 117976=MTCC 13671)	4	[9]
*Microvirga terrestris* Damdintogtokh *et al*. 2022, 5^3^	sp. nov.	BT290 (=KCTC 72367=NBRC 114844)	39	[79]
*Mixta hanseatica* Both *et al*. 2023, 3^3,41^	sp. nov.	X22927 (=DSM 115429=NCTC 14857)	15	[80]
*Mucilaginibacter cryoferens* Kumar *et al*. 2025, 11^3^	sp. nov.	FT3.2 (=DSM 119439=HAMBI 3818)^39^	3	[81]
*Mucilaginibacter empetricola* Kumar *et al*. 2025, 10^3^	sp. nov.	X4EP1 (=DSM 119437=HAMBI 3825)^39^	3	[81]
*Mucilaginibacter formosus* Huq *et al*. 2019, 519	sp. nov.	MAH-5 (=CGMCC 1.16489=KACC 19291)	52	[82]
*Mucilaginibacter geliditolerans* Kumar *et al*. 2025, 10^3^	sp. nov.	X5P1 (=DSM 119435=HAMBI 3824)^39^	3	[81]
*Mucilaginibacter saanensis* Kumar *et al*. 2025, 10^3^	sp. nov.	SP1R1 (=DSM 119438=HAMBI 3819)^39^	3	[81]
*Mucilaginibacter tundrae* Kumar *et al*. 2025, 10^3^	sp. nov.	E4BP6 (=DSM 119436=HAMBI 3826)^39^	3	[81]
*Myceligenerans salitolerans* Guan *et al*. 2013, 150	sp. nov.	XHU 5031 (=CCTCC AB 2012908=KCTC 29128)	58	[83]
*Mycobacterium appelbergii* Roxo *et al*. 2025, 10^3,42^	sp. nov.	21AC1 (=BCCM/ITM 501212=DSM 113570)	95	[84]
*Natronomonas amylolytica* Cui *et al*. 2024, 6^3^	sp. nov.	YCN58 (=CGMCC 1.62712=JCM 36683)	145	[46]
*Neobacillus canaveralius* Schultz *et al*. 2025, 13^3^	sp. nov.	3P2-tot-E-2 (=DSM 118040=MTCC 13929)	4	[9]
*Neobacillus phoenicis* Schultz *et al*. 2025, 12^3^	sp. nov.	1P10SD (=DSM 118030=MTCC 13641)	4	[9]
*Nesterenkonia marinintestina* Zhao *et al*. 2024, 7^3^	sp. nov.	GX14115 (=KCTC 49495=MCCC 1K06658)	102	[85]
*Nocardioides bruguierae* Chen *et al*. 2023, 9^3^	sp. nov.	BSK12Z-3 (=CGMCC 4.7709=JCM 34554)	53	[86]
*Nonomuraea marmarensis* Topkara *et al*. 2025, 9^3^	sp. nov.	M3C6 (=CGMCC 4.8035=KCTC 49983)	96	[87]
*Noviherbaspirillum phoenicis* Schultz *et al*. 2025, 12^3^	sp. nov.	1P10PC (=DSM 118057=MTCC 13944)	4	[9]
*Novosphingobium beihaiense* corrig. Hu *et al*. 2023, 1157^43^	sp. nov.	B2638 (=KCTC 72968=MCCC 1K07406)	136	[88]
*Oceanobacillus saliphilus* OuYang *et al*. 2022, 5^3^	sp. nov.	APA_H-1(4) (=GDMCC 1.2239=KCTC 43254)	120	[89]
*Oryzifoliimicrobium* Liu *et al*. 2025, 7^3^	gen. nov.	*Oryzifoliimicrobium ureilyticum*	94	[90]
*Oryzifoliimicrobium ureilyticum* Liu *et al*. 2025, 7^3,44^	sp. nov.	SG148 (=KCTC 8337=MCCC 1K08901)	94	[90]
*Pacificimonas aurantia* corrig. Li *et al*. 2016, 755^45^	sp. nov.	JLT2012 (=CGMCC 1.12399=LMG 27361)	48	[91]
*Paenibacillus albiflavus* Han *et al*. 2021, 4977	sp. nov.	18JY21-1 (=JCM 33183=KCTC 33964)^46^	31	[92]
*Paenibacillus anseongensis* corrig. Huq 2020, 2028^47^	sp. nov.	MAH-34 (=CGMCC 1.16610=KACC 19974)	54	[93]
*Paenibacillus canaveralius* Schultz *et al*. 2025, 9^3^	sp. nov.	1P03SA (=DSM 117982=MTCC 13637)	4	[9]
*Paenibacillus hubeiensis* Kong *et al*. 2025, 12^3^	sp. nov.	ES5-4 (=GDMCC 1.3540=KCTC 43478)	64	[94]
*Paenibacillus jepli* Schultz *et al*. 2025, 11^3^	sp. nov.	1P07SE (=DSM 118005=MTCC 13640)	4	[9]
*Paenibacillus lutrae* Rodríguez *et al*. 2019, 11^3^	sp. nov.	N10 (=CECT 9541=LMG 30535)	135	[95]
*Paenibacillus oryzisoli* Zhang *et al*. 2017, 73	sp. nov.	1ZS3-15 (=ACCC 19783=JCM 30487)	60	[96]
*Paenibacillus tylopili* Kuisiene *et al*. 2008, 436^48^	sp. nov.	MK2 (=DSM 18927=LMG 23975)	105	[97]
*Paenibacillus xylaniclasticus* Tachaapaikoon *et al*. 2012, 398	sp. nov.	TW1 (=DSM 26531=KCTC 13719=NBRC 106381=TISTR 1914)^49^	104	[98]
*Paenilisteria portnoyi* (Carlin *et al*. 2021) Bouznada *et al*. 2025, 24^3^	comb. nov. [basonym: *Listeria portnoyi* Carlin *et al*. 2021]	FSL L7-1582 (=CCUG 74671=LMG 31921)	37	[99]
*Paenilisteria riparia* (den Bakker *et al*. 2014) Bouznada *et al*. 2025, 24^3^	comb. nov. [basonym: *Listeria riparia* den Bakker *et al*. 2014]	FSL S10-1204 (=DSM 26685=LMG 28119)^50^	37	[99]
*Paenilisteria weihenstephanensis* (Lang Halter *et al*. 2013) Bouznada *et al*. 2025, 23^3^	comb. nov. [basonym: *Listeria weihenstephanensis* Lang Halter *et al*. 2013]	DSM 24698 (=CECT 8544)^51^	37	[99]
*Paracoccus sigandri* corrig. Liu *et al*. 2013, 1138^52^	sp. nov.	M26 (=CCTCC AB 2012865=DSM 26381)	57	[100]
*Paracoccus yibinensis* Luo *et al*. 2025, 8^3,4^	sp. nov.	WLY502 (=CICC 25237=JCM 36429)	22	[101]
*Parelusimicrobium* Mies *et al*. 2025, 11^3^	gen. nov.	*Parelusimicrobium proximum*	19	[35]
*Parelusimicrobium proximum* Mies *et al*. 2025, 12^3^	sp. nov.	Dip518 (=DSM 104964=JCM 31829)	19	[35]
*Parerythrobacter aurantius* Liu *et al*. 2025, 8^3^	sp. nov.	1C24 (=GDMCC 1.2450=KCTC 82667)	149	[102]
*Parvibaculum sedimenti* Wang *et al*. 2020, 2061	sp. nov.	HXT-9 (=CCTCC AB 2019273=KCTC 72547)	82	[103]
*Pedobacter namyangjuensis* Kim *et al*. 2013, 29^4^	sp. nov.	5G38 (=KACC 13938=NBRC 107692)	133	[104]
*Pedobacter polysacchareus* Wang *et al*. 2023, 8^3^	sp. nov.	ZS13-49 (=CCTCC AB 2019394=KCTC 72824)	73	[105]
*Pedobacter zeaxanthinifaciens* (Asker *et al*. 2008) Kim *et al*. 2013, 29^53^	comb. nov. [basonym: *Nubsella zeaxanthinifaciens* Asker *et al*. 2008]	TDMA-5 (=CCUG 54348=KACC 14260=NBRC 102579)	133	[104]
*Peribacillus phoenicis* Schultz *et al*. 2025, 10^3^	sp. nov.	1P06PB (=DSM 117992=MTCC 13942)	4	[9]
*Petralouisia* Afrizal *et al*. 2022, 21^3^	gen. nov.	*Petralouisia muris*	91	[106]
*Petralouisia muris* Afrizal *et al*. 2022, 21^3^	sp. nov.	NM01_1-7b (=DSM 110156=JCM 37187)	91	[106]
*Phocaeicola acetigenes* Do *et al*. 2024, 7^3^	sp. nov.	KGMB11183 (=JCM 35696=KCTC 25284)	123	[107]
*Phycobium* Kim *et al*. 2025, 9^3^	gen. nov.	*Phycobium rhodophyticola*	40	[6]
*Phycobium rhodophyticola* Kim *et al*. 2025, 9^3^	sp. nov.	D3-12 (=JCM 35528=KACC 22712)	40	[6]
*Planococcus beigongshangi* Hao *et al*. 2023, 8^3^	sp. nov.	REN8 (=GDMCC 1.2213=JCM 33964)	125	[108]
*Planococcus beijingensis* Hao *et al*. 2023, 9^3^	sp. nov.	REN14 (=GDMCC 1.2209=JCM 34410)	125	[108]
*Planococcus soli* Zhang *et al*. 2021, 1113	sp. nov.	C23 (=CGMCC 1.15115=KCTC 33644)	51	[109]
*Proteus appendicitidis* He *et al*. 2024, 6^3,54^	sp. nov.	HZ0627 (=CCTCC AB 2022380=KCTC 92986)	81	[110]
*Pseudidiomarina fusca* Wang *et al*. 2024, 9^3^	sp. nov.	GXY010 (=JCM 35760=KCTC 92693)^55^	106	[111]
*Pseudomarimonas salicorniae* Jeong *et al*. 2024, 6^3^	sp. nov.	CAU 1642 (=KCTC 92084=MCCC 1K07085)	143	[112]
*Pseudomonas jilinensis* Wang *et al*. 2020, 693	sp. nov.	JS15-10A1 (=CGMCC 1.16072=LMG 30036)	98	[113]
*Pseudomonas sputi* Tohya *et al*. 2023, 1^3^	sp. nov.	BML-PP014 (=JCM 34577=LMG 32246)	97	[114]
*Pseudoruegeria limi* Lee *et al*. 2014, 992	sp. nov.	D-17 (=JCM 19487=KCTC 32460)	5	[115]
*Pseudothermotoga caldifontis* (Mori *et al*. 2014) Belahbib *et al*. 2018, 561	comb. nov. [basonym: *Thermotoga caldifontis* Mori *et al*. 2014]	AZM44c09 (=DSM 23272=NBRC 106116)	85	[116]
*Pyranulibacter* Podosokorskaya *et al*. 2025, 8^3^	gen. nov.	*Pyranulibacter aquaticus*	62	[117]
*Pyranulibacter aquaticus* Podosokorskaya *et al*. 2025, 8^3^	sp. nov.	4301-Me (=BIM B-2047=CGMCC 1.18061=UQM 41592=VKM B-3822)^56^	62	[117]
*Rhizobium album* Hang *et al*. 2019, 325	sp. nov.	NS-104 (=CCTCC AB 2017250=KCTC 62327)	79	[118]
*Rhizobium mulingense* Liu *et al*. 2025, 8^3^	sp. nov.	MC63 (=CCTCC AB 2024025=JCM 36652)	16	[119]
*Rhodococcus kronopolitis* Liu *et al*. 2014, 1210^57^	sp. nov.	NEAU-ML12 (=CGMCC 4.7145=DSM 46702)	13	[120]
*Robertmurraya phoenicis* Schultz *et al*. 2025, 13^3^	sp. nov.	2P01SA (=DSM 118031=MTCC 13642)	4	[9]
*Roseomonas marmotae* Zhu *et al*. 2022, 144	sp. nov.	1318 (=GDMCC 1.1781=JCM 34188)	65	[121]
*Rossellomorea yichunensis* Kong *et al*. 2025, 9^3^	sp. nov.	YC4-1 (=GDMCC 1.3541=KCTC 43479)	63	[122]
*Rufibacter soli* Jang *et al*. 2016, 637	sp. nov.	FJY1 (=JCM 31024=KCTC 42815)	116	[123]
*Salinicola zeshunii* Cao *et al*. 2013, 195	sp. nov.	N4 (=CCTCC AB 2012912=KACC 16602)	44	[124]
*Shimazuella soli* Jin *et al*. 2022, 9^3^	sp. nov.	AN120528 (=DSM 103571=KCTC 39810)	109	[125]
*Sphingomicrobium aquimarinum* Zhang *et al*. 2025, 11^3,58^	sp. nov.	XHP0235 (=GDMCC 1.3093=JCM 35574=MCCC 1K07532)	1	[126]
*Sphingomicrobium maritimum* Zhang *et al*. 2025, 11^3,58^	sp. nov.	XHP0239 (=GDMCC 1.3086=JCM 35575=MCCC 1K07530)	1	[126]
*Sphingomonas caseinilytica* corrig. Siddiqi *et al*. 2023, 4^3,59^	sp. nov.	NSE70-1 (=LMG 32495=KACC 22411)	138	[127]
*Sphingomonas humi* Yi *et al*. 2010, 167	sp. nov.	PB323 (=JCM 16603=KCTC 12341)^60^	7	[128]
*Sphingomonas hunanensis* Chen *et al*. 2011, 757	sp. nov.	JSM 083058 (=CCTCC AA 209011=DSM 22213)	43	[129]
*Streptococcus halichoeri* subsp. *halichoeri* (Lawson *et al*. 2004) Shewmaker *et al*. 2016, 743	comb. nov. [basonym: *Streptococcus halichoeri* Lawson *et al*. 2004]	M512/02/1 (=CCUG 48324=CIP 108195=DSM 17028)^61^	29	[130]
*Streptococcus halichoeri* subsp. *hominis* Shewmaker *et al*. 2016, 743	subsp. nov.	SS1844 (=CCUG 67100=LMG 28801)	29	[130]
*Streptococcus taonis* Lee *et al*. 2024, 5^3^	sp. nov.	ST22-14 (=BCRC 81402=NBRC 116002)	144	[131]
*Streptomonospora litoralis* Khodamoradi *et al*. 2021, 1493^62^	sp. nov.	M2 (=DSM 106425=NCCB 100650)	147	[132]
*Streptomyces buecherae* Hamm *et al*. 2020, 2220^63^	sp. nov.	AC541 (=ATCC TSD-201=JCM 34263)	38	[133]
*Streptomyces chengbuensis* Zheng *et al*. 2024, 574^4,49^	sp. nov.	HUAS CB01 (=JCM 36277=MCCC 1K08666)	128	[134]
*Streptomyces ferrugineus* Ruan *et al*. 2015, 43	sp. nov.	HV38 (=CCTCC AA 2014009=DSM 42152)	68	[135]
*Streptomyces gilvus* Tangjitjaroenkun *et al*. 2025, 10^3^	sp. nov.	CME 23 (=NBRC 115859=TBRC 15810)	146	[136]
*Streptomyces luozhongensis* Zhang *et al*. 2017, 200^64^	sp. nov.	TRM 49605 (=CCTCC AA 2015026=KCTC 39666)	137	[137]
*Streptomyces piniterrae* Zhuang *et al*. 2020, 7^3^	sp. nov.	jys28 (=CCTCC AA 2018051=DSM 109823)	67	[138]
*Streptomyces polaris* Kamjam *et al*. 2019, 384	sp. nov.	ZLN81 (=CCTCC AA 2018010=DSM 107255)	66	[139]
*Streptomyces polyasparticus* Liu *et al*. 2021, 783	sp. nov.	TRM66268-LWL (=CCTCC AA 2020003=LMG 32106)	83	[140]
*Streptomyces septentrionalis* Kamjam *et al*. 2019, 384	sp. nov.	ZLN712 (=CCTCC AA 2018011=DSM 107266)	66	[139]
*Streptomyces tritrimontium* Bielańska *et al*. 2025, 8^3^	sp. nov.	2.9 (=DSM 119353=PCM 3548)	17	[141]
*Streptomyces typhae* Peng *et al*. 2021, 831	sp. nov.	p1417 (=CCTCC AA 2019091=DSM 110636)^65^	72	[142]
*Streptomyces urticae* Piao *et al*. 2018, 1841	sp. nov.	NEAU-PCY-1 (=CCTCC AA 2017015=DSM 105115)	70	[143]
*Streptosporangium kronopolitis* Ma *et al*. 2015, 1496^66^	sp. nov.	NEAU-ML10 (=CGMCC 4.7153=DSM 46720)	148	[144]
*Sulfurospirillum carboxydivorans* corrig. Jensen and Finster 2005, 350^67^	sp. nov.	MV (=ATCC BAA-937=DSM 16295)	8	[145]
*Thalassovivens* Lanclos *et al*. 2025, 11^3^	gen. nov.	*Thalassovivens spotae*	20	[146]
*Thalassovivens spotae* Lanclos *et al*. 2025, 11^3^	sp. nov.	US3C007 (=ATCC TSD-433=DSM 119208)^68^	20	[146]
*Thauera sedimentorum* Baek *et al*. 2022, 6^3^	sp. nov.	CAU 1555 (=KCTC 72546=MCCC 1K04065)	130	[147]
*Thermalkalibacillus* Zhao *et al*. 2006, 343^69^	gen. nov.	*Thermalkalibacillus uzonensis*	25	[148]
*Thermalkalibacillus uzonensis* Zhao *et al*. 2006, 343^70^	sp. nov.	JW/WZ-YB58 (=ATCC BAA-1258=DSM 17740)	25	[148]
*Tropicibacter oceani* Zhou *et al*. 2023, 1342	sp. nov.	YMD87 (=KCTC 92856=MCCC 1K08473)	121	[149]
*Umezawaea beigongshangensis* Ren *et al*. 2021, 4130	sp. nov.	REN6 (=CGMCC 19205=JCM 33954)	56	[150]

For references to Validation Lists 1–71, see Int J Syst Bacteriol 49 (1999) 1325. Lists 72–197 were published in Int J Syst Evol Microbiol 50 (2000) 3, 423, 949, 1415, 1699, 1953; and 51 (2001) 1, 263, 793, 1229, 1619, 1945; and 52 (2002) 3, 685, 1075, 1437, 1915; and 53 (2003) 1, 373, 627, 935, 1219, 1701; and 54 (2004) 1, 307, 631, 1005, 1425, 1909; and 55 (2005) 1, 547, 983, 1395, 1743, 2235; and 56 (2006) 1, 499, 925, 1459, 2025, 2507; and 57 (2007) 1, 433, 893, 1371, 1933, 2449; and 58 (2008) 1, 529, 1057, 1511, 1993, 2471; and 59 (2009) 1, 451, 923, 1555, 2129, 2647; and 60 (2010) 1, 469, 1009, 1477, 1985, 2509; 61 (2011) 1, 475,1011, 1499, 2025, 2563; and 62 (2012) 1, 473, 1017, 1443, 2045, 2549; and 63 (2013) 1, 797, 1577, 2365, 3131, 3931; and 64 (2014) 1, 693, 1455, 2184, 3603; and 65 (2015), 1, 741, 1112, 2017, 2777, 3767; and 66 (2016) 1, 1603, 1913, 2463, 3761, 4299; and 67 (2017) 1, 529, 1095, 2075, 3140, 4291; and 68 (2018), 1, 693, 1411, 2130, 2707, 3379; and 69 (2019), 5, 597, 1247, 1844, 2627, 3313, and 70 (2020) 1, 1443, 2960, 4043, 4844, 5596. Lists 197–225 were published in Int J Syst Evol Microbiol 71 (2021) 004600, 004688, 004773, 004846, 004943, 005096; and 72 (2022) 005167, 005260, 005331, 005422, 005517, 005592; and 73 (2023) 005709, 005812, 005845, 005931, 005997, 006080; and 74 (2024) 006173, 006229, 006275, 006398, 006452, 006501; and 75 (2025) 006562, 006640, 006699, 006863.

1Abbreviations of culture collections cited in this list can be found at https://lpsn.dsmz.de/text/collections.

2Priority number assigned according to the date the documentation and request for validation are received.

3The journal in which the name was effectively published does not have continuous pages.

4The etymology must state N.L. masc. adj. instead of N.L. fem. adj.

5The syllabification and etymology must state mont.re.al.en’sis. N.L. masc. adj. *montrealensis*.

6The list editors have corrected *euxinus* to *euxina* (eu.xi’na. L. fem. adj. *euxina*, hospitable).

7Created automatically with the creation of subsp. *kamchatkensis*.

8Homotypic synonym of *Alkalihalobacillus hunanensis *Patel and Gupta 2020 and of *Shouchella hunanensis* (Patel and Gupta 2020) Joshi *et al.* 2022.

9An erratum has been published, correcting JCM 36016 to 36011.

10The syllabification and etymology must state Bi.na.ri.i.mo’nas. L. masc. adj. *binarius*, that contains or consists of two; Gr. fem. n. *monas*.

11The preferred syllabification is pa.ci’fi.ca.

12The list editors have corrected *hydrothermale* to *hydrothermalis* (hy.dro.ther.ma’lis. Gr. neut. n. *hydor*, water; Gr. masc. adj. thermos, hot; N.L. fem. adj. *hydrothermalis*, pertaining to hot water, hydrothermal).

13The effective publication states that the type strain was also deposited as KCOM 1579 and KCCM 42841, but no documentation was supplied.

14The effective publication states that the type strain was also deposited as NBRC 115006, but no documentation was supplied.

15The authors gave DSMZ 113825 instead of DSM 113825.

16The list editors have corrected the epithet to *alearensis *(a.le.ar.en’sis. N.L. masc. adj. *alearensis*, referring to Alear City, …).

17The preferred etymology is N.L. masc. adj. instead of N.L. adj.

18 The effective publication states that the type strain was also deposited as HNMC11 208, but no documentation was supplied.

19 The list editors have corrected the epithet to *candida *(can’di.da. L. fem. adj. *candida*, white).

20The protologue and abstract state TSD-314 instead of ATCC TSD-314.

21The list editors have corrected *sahariens* to *saharensis *(sa.ha.ren’sis. N.L. masc. adj. *saharensis*, pertaining to the Sahara …).

22The strain number was given as HAN23 in the protologue, as HAN-23 and HAN 23 elsewhere in the article.

23Homotypic synonym of *Desulfocicer niacini *(Kuever et al. 2006) Galushko and Kuever 2021 and of *Desulfobacterium niacini *Kuever et al. 2006.

24The list editors have corrected *caldoproteolyticus* to *caldiproteolyticus* (cal.di.pro.te.o.ly’ti.cus. L. masc. adj. *caldus*, hot; N.L. masc. adj. *proteolyticus*, proteolytic; N.L. masc. adj. *caldiproteolyticus*, hot and protein-degrading); homotypic synonym of *Anoxybacillus caldiproteolyticus* Coorevits *et al*. 2012.

25The etymology must state N.L. masc. adj. instead of M.L. gen. n. adj.

26Created automatically with the creation of subsp. *stromboliensis*.

27The etymology must state N.L. masc. adj. instead of N.L. neut. adj.

28The etymology must state Gr. masc. n. *grylos*, a pig.

29The list editors have corrected the epithet to *aquisgranense *(a.quis.gra.nen’se. L. neut. adj. *aquisgranense*, pertaining to *Aachen* …).

30The list editors have corrected *Intestinicryptomonaceae* to *Intestinicryptomonadaceae* (In.tes.ti.ni.cryp.to.mo.na.da.ce’ae. N.L. fem. n. *Intestinicryptomonas*, a bacterial genus name; -*aceae*, ending to denote a family; N.L. fem. pl. n. *Intestinicryptomonadaceae*, the *Intestinicryptomonas* family).

31The list editors have corrected *Jejubacter *to *Jejuibacter *(Je.ju.i.bac’ter. N.L. masc. n. *bacter*, a rod; N.L. masc. n. *Jejuibacter*, a rod from Jeju-do, an island in Korea).

32Given as *Kitasatosporia*, the generic name originally proposed in 1982 by Ōmura *et al*., instead of *Kitasatospora* as corrected by Zhang *et al.* in 1997.

33The effective publication states that the type strain was also deposited as IAM 13638, but no documentation was supplied.

34The effective publication states that the type strain was also deposited as FERM 9000 and IAM 13637, but no documentation was supplied.

35The effective publication states that the type strain was also deposited as UQM 41800, but no documentation was supplied.

36The list editors have corrected the epithet *piscensis *to *piscis* (pis’cis. L. gen. n. *piscis*, of a fish).

37The DSM number was given in the abstract as DSM 26805; in the protologue, it was erroneously given as DSM=26,804.

38The protologue heading must state *Marinobacter squalenivorans* instead of *M. squalenivorans*, and the etymology must be as follows: squa.le.ni.vo’rans. N.L. neut. n. *squalenum*, squalene; L. pres. part. *vorans*, devouring, consuming; N.L. masc. part. adj. *squalenivorans*, squalene-devouring.

39DSM was given as DSMZ in the culture collection number in the protologue.

40The syllabification and etymology must state what.ton.en’se. N.L. neut. adj. *whartonense*, pertaining to Wharton State Park.

41The syllabification and etymology must state han.se.a’ti.ca. M.L. fem. adj. *hanseatica*, pertaining to the north-German medieval commercial alliance ‘Hanse’ of which Hansestadt Hamburg, where the type strain was isolated, was a member.

42The etymology must state N.L. gen. n. instead of N.L. adj.

43The list editors have corrected the epithet to *beihaiense* (bei.hai.en’se. N.L. neut. adj. *beihaiense*, …).

44The syllabification must state u.re.i.ly’ti.cum. The epithet was also misspelled *ureilyticus* in the title and elsewhere in the article.

45The list editors have corrected the epithet to *aurantia* (au.ran’ti.a. N.L. fem. adj. *aurantia*, orange-coloured).

46In the abstract, KCTC 33964 was erroneously given as KCTC 3396.

47The list editors have corrected *anseongense* to *anseongensis* (an.seong.en’sis. N.L. masc. adj. *anseongensis*, …).

48The preferred syllabification and etymology are ty.lo’pi.li. (N.L. gen. n. *tylopili*, of the fungal genus *Tylopilus*).

49The culture collection numbers are given in the abstract, not in the protologue.

50The effective publication states that the type strain was also deposited as BEI NR-42634, but no documentation was supplied.

51The effective publication states that the type strain was also deposited as LMG 26374 and WS 4560, but no documentation was supplied.

52The list editors have corrected *siganidrum* to *sigani* (si.ga’ni. N.L. gen. n. *sigani*, of the fish genus *Siganus*).

53Syllabification and etymology must be added as follows: ze.a.xan.thi.ni.fa’ci.ens. N.L. neut. n. *zeaxanthinum*, zeaxanthin; L. pres. part. *faciens*, making/producing; N.L. fem. part. adj. *zeaxanthinifaciens*, zeaxanthin-producing.

54The protologue heading must state *Proteus* instead of *P.*

55The effective publication states that the type strain was also deposited as MCCC M28199, but no documentation was supplied.

56The authors gave B-3822 instead of VKM B-3822.

57The etymology must state N.L. gen. n. *kronopolitis*, of the insect genus *Kronopolites*.

58The etymology must state adj. instead of Adj.

59The list editors have corrected the epithet to *caseinilytica* (ca.se.i.ni.ly’ti.ca. N.L. neut. n. *caseinum*, casein; N.L. masc. adj. *lyticus* (from Gr. masc. adj. *lytikos*), able to dissolve; N.L. fem. adj. *caseinilytica*, casein-dissolving).

60The effective publication states that the type strain was also deposited as KEMB 9004-003, but no documentation was supplied.

61The effective publication states that the type strain was also deposited as CCUG 48325, but no documentation was supplied.

62C L. fem. adj. instead of L. masc. adj.

63The preferred syllabification and etymology are bue’che.rae. N.L. gen. n. *buecherae*, named in honor of Debbie Buecher.

64The etymology must state N.L. masc. adj. instead of N.L. Masc. adj.

65The protologue erroneously gave CCTCC AA 2,019,091 and DSM 110,636.

66The preferred etymology is N.L. gen. n. *kronopolitis*, of the insect *Kronopolites svenhedind*.

67The list editors have corrected the epithet to *carboxydivorans* (car.bo.xy.di.vo’rans. N.L. neut. n. *carboxydum*, carbon monoxide; L. pres. part. *vorans*, devouring, consuming; N.L. neut. part. adj. *carboxydivorans*, …).

68The effective publication states that the type strain was also deposited as NCMA B160, but no documentation was supplied.

69The preferred syllabification is Therm.al.ka.li.ba.cil’lus.

70The protologue heading must state *Thermalkalibacillus uzonensis* instead of *T. uzonensis*.

## Corrigenda to Validation List no. 224 [151]

The name *Aeromonas mytilicola* subsp. *aquatica* Ajmi *et al*. 2025 is illegitimate because of the presence of *Aeromonas aquatica* Beaz-Hidalgo *et al*. 2015 (Rule 53).

The type strain of *Streptomyces asiaticus* subsp. *asiaticus* (Sembiring *et al*. 2001) Nouioui *et al*. 2025 must be given as A14P1 (=DSM 41761=JCM 11443=NBRC 100774=NCIMB 13675); A10598 (=ATCC 15166=DSM 41524) is the type strain of *S. asiaticus* subsp. *ignotus* Nouioui *et al*. 2024.

The name *Streptomyces bugieae* Nouioui *et al*. 2024 was incorrectly given as *Streptomyces bugiae* in the Validation List.

## Abbreviations

IJSEM: International Journal of Systematic and Evolutionary Microbiology
